# Chitosan Micro/Nanocapsules in Action: Linking Design, Production, and Therapeutic Application

**DOI:** 10.3390/molecules30020252

**Published:** 2025-01-10

**Authors:** Yaride Pérez-Pacheco, Bartosz Tylkowski, Ricard García-Valls

**Affiliations:** 1 Department of Chemical Engineering, Universitat Rovira i Virgili, Av. Països Catalans 26, Campus Sescelades, 43007 Tarragona, Spain; yaride.perez@urv.cat (Y.P.-P.); bartosz.tylkowski@eurecat.org (B.T.); 2 Eurecat, Centre Tecnològic de Catalunya, Chemical Technologies Unit, Marcel_lí Domingo s/n, 43007 Tarragona, Spain; 3 Faculty of Health Science, Collegium Medicum in Bydgoszcz, Nicolaus Copernicus University in Toruń, ul. Sklodowskiej Curie 9, 85-094 Bydgoszcz, Poland

**Keywords:** chitosan, chitosan derivatives, chitosan capsules

## Abstract

pH sensitivity of chitosan allows for precise phase transitions in acidic environments, controlling swelling and shrinking, making chitosan suitable for drug delivery systems. pH transitions are modulated by the presence of cross-linkers by the functionalization of the chitosan chain. This review relays a summary of chitosan functionalization and tailoring to optimize drug release. The potential to customize chitosan for different environments and therapeutic uses introduces opportunities for drug encapsulation and release. The focus on improving drug encapsulation and sustained release in specific tissues is an advanced interpretation, reflecting the evolving role of chitosan in achieving targeted and more efficient therapeutic outcomes. This review describes strategies to improve solubility and stability and ensure the controlled release of encapsulated drugs. The discussion on optimizing factors like cross-linking density, particle size, and pH for controlled drug release introduces a deeper understanding of how to achieve specific therapeutic effects. These strategies represent a refined approach to designing chitosan-based systems, pushing the boundaries of sustained release technologies and offering new avenues for precise drug delivery profiles.

## 1. Chitosan and pH Sensitivity

**Figure 1:**
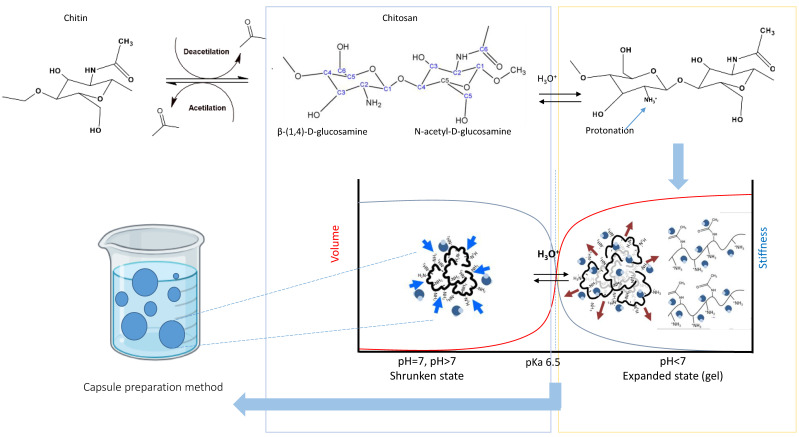
Schematic representation of chitosan-based capsule preparation and pH-responsive behavior.

Chitosan (CS) is a cationic polysaccharide product from the deacetylation of chitin. Chitin is insoluble and chemically inert and abundantly found in fungi and marine crustaceans [1]. When chitin is treated with concentrated alkali solutions, the acetamide groups (-NH-CO-CH_3_) are deacetylated into amino groups (-NH_2_) to produce chitosan as seen in Figure 1, ranging from 66% to 95% deacetylated with average molecular weight from 3800 to 300,000 Daltons [2,3]. Chitosan remains insoluble in water at neutral or basic pH due to the presence of free amino groups (-NH_2_). However, in acidic environments, these amino groups undergo protonation (-NH_3_^+^), making chitosan water-soluble and imparting pH-sensitive properties as seen in Figure 1. This pH sensitivity triggers a phase transition, changing its volume and swelling behavior in response to pH changes. In acidic conditions, the protonated amine groups carry a positive charge, leading to electrostatic repulsion between the chitosan chains. As these chains repel one another, water easily infiltrates the network, causing the material to swell significantly. When the pH rises, the amine groups deprotonate, losing protons and becoming neutral. This reduces the charge density within the chains, weakening electrostatic repulsion, and causing the chitosan network to shrink as it collapses [4]. Consequently, the apparent pKa of chitosan, the pH at which half of its amine groups are ionized, is critical to this phase transition. Below the pKa, chitosan remains protonated and swollen due to strong repulsive forces. As the pH approaches the pKa, ionization increases, reaching maximum repulsion before all ionizable groups are fully protonated, marking the end of the swelling process [5].

Chitosan exists in two main phases, determined by the dominant interactions. In the chitosan–chitosan interaction phase, the interactions between chitosan molecules are stronger than those with the solvent, resulting in a gel-like structure where the polymer shrinks and exhibits maximum hydrophobicity. In contrast, in the solvent–chitosan interaction phase, solvent molecules interact more strongly with chitosan than the polymer molecules do with each other, leading to swelling and the polymer achieving its highest hydrophilicity and expansion in volume [6].

This swelling behavior can be fine-tuned by modifying the structure of chitosan. For instance, incorporating hydrophobic groups into the chitosan backbone can shift the pKa, enabling the phase transition to occur at different pH levels. This adaptability makes chitosan useful for applications such as drug delivery in environments with varying pH values. Moreover, the degree of swelling or shrinking is also influenced by factors such as the ionic strength of the environment, the presence of counterions, and the types of functional groups present in the chitosan structure. These factors affect the electrostatic interactions within the polymer network, consequently modulating its overall behavior [5,6].

## 2. Chitosan Attractiveness for Medical Applications in Drug Delivery and Devices

The versatility of chitosan as a drug delivery material stems from its ability to control key properties, such as the degree of deacetylation and molecular weight. The degree of deacetylation affects the balance between hydrophobic interactions and hydrogen bonding, while the molecular mass influences its protonation constant (pKa), which can range from 6.51 to 6.39 as the molecular mass changes from 1370 to 60 kDa [7]. These tunable properties make chitosan highly suitable for drug delivery systems [7]. Furthermore, chitosan can be functionalized [8,9,10,11], enabling the efficient encapsulation of drugs [12], undependably of the drug properties, like anticancer drugs [13,14,15,16], and essential oils [17,18,19,20]. Chitosan ability to produce micro-nanoparticles as loaded capsules for controlled and targeted drug delivery is particularly useful for enhancing the dissolution rate of poorly soluble drugs for targeting specific tissues or cells [21].

**Figure 2:**
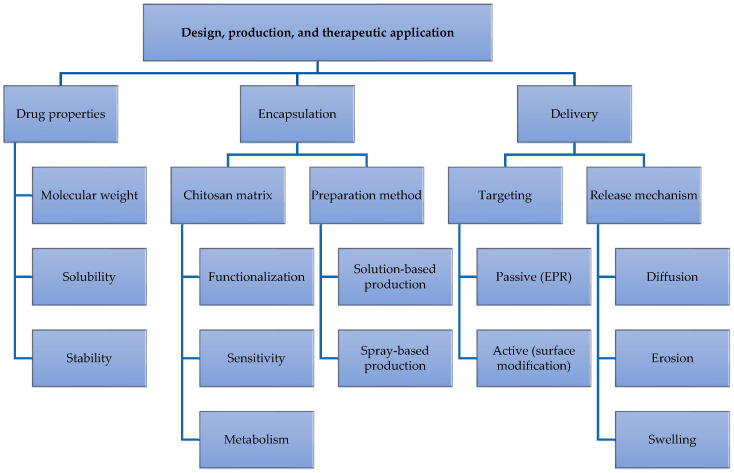
Design, production, and therapeutic application of chitosan.

Chitosan mucoadhesive properties make it particularly effective for targeting mucosal tissues [22,23], such as nasal [24], ocular [25], and gastrointestinal [26], dermal [27,28], and tumoral [13,29,30,31,32] sites. Chitosan adhesion to these surfaces extends contact time, allowing sustained drug release and improved absorption. Chitosan nanocapsules can also enter into the cells by adsorptive endocytosis [33]. For example, chitosan internalization can be modulated by its molecular weight and degree of deacetylation as tested in A549 cells, a type of lung cancer cell line; uptake fell by 26% when molecular mass was decreased from 213.000 to 10.000 Da, and by 41% when the degree of deacetylation was lowered from 88% to 46% [34]. Chitosan micro/nanocapsules have been proven to have good biocompatibility and no cytotoxicity to both human and mouse tissue cell culture [35] but clinical trials are still under investigation (Table 1). Furthermore, chitosan has intrinsic antimicrobial properties useful in preventing infections related to implanted drug delivery devices [36]. In conclusion, chitosan properties make it an ideal material for the design, production, and therapeutic application of advanced drug delivery systems as summarized in Figure 2.

Ongoing research focuses on optimizing chitosan properties for more effective drug delivery, encapsulation efficiency, controlled drug release, and targeted therapy. With continuous developments, chitosan is expected to play a vital role in the future of drug delivery designs. For example, combined physicochemical and pH-responsive properties of chitosan nanoparticles have been shown to enhance doxorubicin cell internalization in a tumor cell model [13]. It has been demonstrated that the use of chitosan nanoparticles increased the intestinal permeation of doxorubicin to nearly 90%, presenting a promising platform for the oral delivery of drugs that are typically poorly absorbed [14]. Also, enteric-coated chitosan micro/nanocapsules were shown to travel through the gastrointestinal tract and reach the large intestine within 1 to 3 h, where they degraded in the colon. This offers potential for site-specific delivery to the lower gastrointestinal tract, an important feature for treating conditions like colon cancer or inflammatory bowel disease [37].

**Figure 3:**
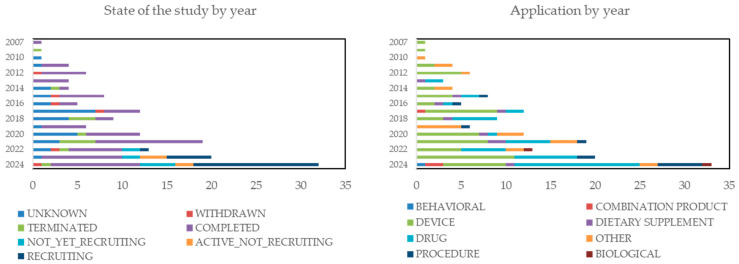
Current clinical trials of chitosan-based studies (https://www.clinicaltrials.gov/search?term=Chitosan&viewType=Table accessed on 5 September 2024).

Translating chitosan-based research into clinical practice involves rigorous preclinical and clinical trials to ensure the safety, efficacy, and regulatory compliance of the drug formulations. Many chitosan-based materials, as referenced in Table 1, have been tested through in vitro and in vivo experiments and have demonstrated strong biocompatibility in animal models. Since 2007, there have been 155 clinical studies involving chitosan-based medical applications, though only 13 have reported results. Among these, 44 applications of chitosan for micro/nanocapsule use, drug support, or encapsulation have been tested as shown in Figure 3.

Particularly, two successful phase III trials have provided results. The first study (NCT03186261) demonstrated the antibacterial effectiveness of a nanosilver fluoride solution composed of silver nanoparticles, chitosan, and fluoride in treating carious molars. The second study (NCT02789033) showed the efficacy of a combination of isosorbide dinitrate spray and chitosan in treating diabetic foot ulcers. These promising outcomes underscore the potential of chitosan in clinical applications.

However, before new chitosan-based drugs can reach the market, regulatory agencies like the U.S. Food and Drug Administration (FDA) and the European Medicines Agency (EMA) require extensive documentation to ensure that these treatments meet all safety and efficacy standards. As research continues to progress, chitosan is expected to play a pivotal role in the future of drug delivery, offering innovative solutions for drug delivery, targeted therapies, and other medical application standards [38].

## 3. Chitosan Functionalization

To enhance the effectiveness of chitosan as a drug delivery system, it is crucial to explore the opportunities for optimizing its physicochemical properties, which can significantly improve targeting and delivery efficiency. By fine-tuning these characteristics, chitosan can be better tailored, or chemically functionalized for specific therapeutic applications, ensuring more precise and effective treatment outcomes.

The unique chemical structure of chitosan and its reactive functional groups allow for a variety of different reactions on primary amino groups at the C2 position and primary and secondary hydroxyl groups at C6 and C3 positions, respectively, in β-(1,4)-D-glucosamine as seen in Figure 1. These groups allow chemical modifications. The amino groups at C2 generally are more reactive than the hydroxyl groups, with the reactivity order being C2-NH_2_ > C6-OH > C3-OH [8]. Also, these chitosan amino groups (at C2 position) are nucleophilic and reactive at higher pH values [39].

These modifications can enhance solubility in different media, expanding its applications and enhancing solubility of hydrophobic drugs. Chitosan derivatives present amphiphilic characteristics, stabilized by hydrophobic interactions. Chitosan derivatives are being developed to address specific therapeutic needs by modifying its properties. These derivatives can reduce matrix hydration and enhance network stabilization through hydrophobic interactions [39], improve solubility [40], reduce stiffness [41], and impart responsive characteristics such as thermo-responsiveness [42], photo-responsiveness [43], and pH/redox responsiveness [44]. Additional modifications include magnetic responsiveness [45] and dual enzyme/pH responsiveness [21]. These tailored properties enable the formation of microstructures like hydrogels [46], while improving the biological performance of the material for various applications [39,47,48], including the integration of specific responsive molecules.

**Figure 4:**
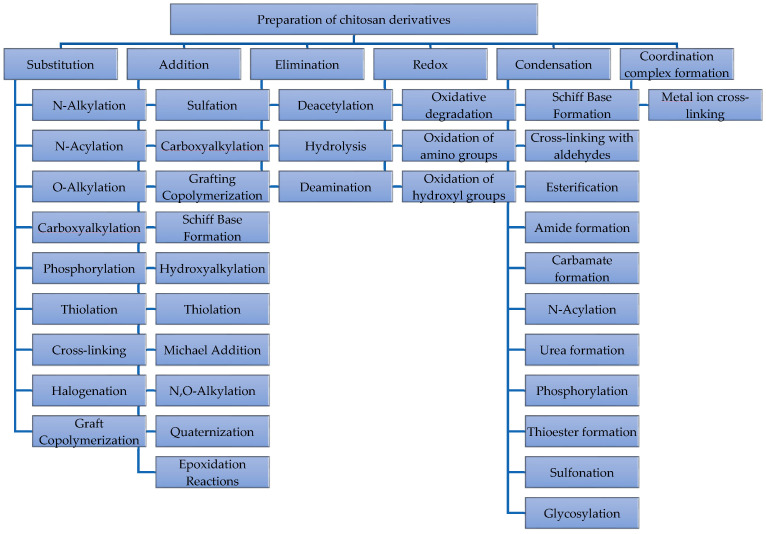
Chemical preparation of chitosan derivatives.

The functional groups of chitosan that enable reactions of substitution, addition, elimination, redox, condensation, and coordination complex formation, according to Figure 4, are named as follows:

- Initially, substitution reactions carried out with chitosan allow the introduction of various functional groups like the following: In N-acylation, the hydrogen of the amino groups is substituted by acyl groups (-COR) using acyl chlorides, acid anhydrides, or carboxylic acids [8]. N-alkylation is a replacement of hydrogen in the amino groups with alkyl groups using alkyl halides (e.g., methyl iodide, ethyl bromide) or other alkylating agents [49]. O-alkylation is the substitution of hydrogen in hydroxyl groups with alkyl groups, modifying the structure via the hydroxyl groups. Sulfation is the introduction of sulfate groups (-SO_4_^2−^) by reacting hydroxyl and amino groups of chitosan with sulfuric acid derivatives such as chlorosulfonic acid or sulfur trioxide. Phosphorylation is a substitution of hydroxyl groups with phosphate groups (-PO_4_^3−^), using reagents like phosphorus oxychloride or phosphoric acid [50]. Carboxymethylation is the introduction of carboxymethyl groups (-CH_2_COOH) by substituting the amino or hydroxyl groups using monochloroacetic acid [21,51]. The quaternization of the amino group with quaternary ammonium groups (-NR_4_^+^) using alkylating agents like methyl iodide in the presence of a tertiary amine increases the positive charge on chitosan [52]. Halogenation replaces hydrogen atoms in the amino or hydroxyl groups with halogens, producing N-halamine-based chitosan through chlorine bleach treatment [36,53]. Thiol (-SH) addition (thiolation) to amino or hydroxyl groups is by sulfhydryl-bearing agents attached covalently to the primary amino group by amide or amidine bonds [44,54,55,56]. Aminoalkylation substitutes the amino or hydroxyl groups with aminoalkyl groups, using reagents like aminoalkyl halides [57]. Benzylation is the substitution with benzyl groups (-C_6_H_5_CH_2_), typically improving the hydrophobicity or interaction with aromatic compounds [58].

- Secondly, addition reactions like the addition of sulfate groups (-SO_4_^2−^) to the amino groups, forming sulfamate products (−NH-SO_3_^−^) and sulfonated products (−NH-R-SO_3_^−^), and also the sulfonation reaction, may take place on hydroxyl groups, resulting in sulfated products (−O-SO_3_^−^) [59]. Carboxyalkylation is where carboxyalkyl groups (-COOR) can be added to the amine or hydroxyl positions by using aza-Michael addition and substitution reactions [60,61]. Alkylation is where alkyl groups are introduced by reacting alkyl halides with the amino group. There is also the addition of thiol groups (-SH) to the amino groups. Imine formation is where amino groups react with aldehydes or ketones to form Schiff bases (C=N) [62]. Quaternary ammonium salts react with chitosan to form a cationic polymer [52]. Radical polymerization is used to graft copolymers onto chitosan like N-isopropylacrylamide (NIPAM) derivatives [42,45]. Hydroxyalkyl groups are added to the amino or hydroxyl groups. Michael addition is where a nucleophilic addition occurs at the amino group with electron-deficient double bonds (e.g., acrylates) [61]. The epoxide reaction is where chitosan amino or hydroxyl groups can open epoxide rings, forming covalent attachments [41,48].
- Thirdly, elimination reactions like the deacetylation of acetyl groups (-COCH_3_) from N-acetylglucosamine units result in free amino groups (-NH_2_) and the deamination of amino groups (-NH_2_) under strong acidic or oxidative conditions, leading to the formation of carbonyl (-C=O) or imine (-C=N) groups [8,63].
- Fourth, there are redox reactions like oxidative degradation by using agents like H_2_O_2_, KMnO_4_, 2,2,6,6-Tetramethylpiperidine-1-oxyl, or periodates of either the hydroxyl or amino groups or the 1,4-glycosidic bond of chitosan to depolymerize by free-radical-mediated degradation [64,65]. There is the nitrosation of primary amino groups to nitroso (-NO) or nitro (-NO_2_) groups using nitric or nitrous acid; these nitrosating species attack the amine groups, but not the N-acetyl moieties, and subsequently cleave the β-glycosidic linkages [66]. And there is the oxidation of hydroxyl groups at the C6 position to aldehydes (-CHO) or carboxylic acids (-COOH) with nitrogen oxides generated in situ from a HNO_3_/H_3_PO_4_–NaNO_2_ mixture [48].
- Fifth, there are condensation reactions like imine formation by the reaction of amino groups with aldehydes or ketones by eliminating water [67]. There is esterification by the reaction of the hydroxyl groups with carboxylic acids or their derivatives; the esterification reaction happens in both acidic and basic environments when a –COOH group encounters a hydroxyl group, eliminating water or HCl [32]. There is amide formation by the reaction of the amino groups with carboxylic acids or their derivatives, eliminating water or HCl [11,68]. There is carbamate formation (urethane chitosan—NHCO_2_-NH_4_^+^) by the reaction of amino groups with isocyanates (-N=C=O), eliminating CO_2_ [40]. There is acylation by the reaction of amino groups with fatty acid (C6–C16) chlorides or acyl chlorides or anhydrides, forming amides and eliminating HCl or a carboxylic acid [39]. There is urea formation by the reaction of amino groups with isocyanates to form ureido groups (-NH-C(O)-NH_2_), eliminating CO_2_ [46]. There is the phosphorylation of chitosan with phosphoric acid derivatives to form phosphoesters, eliminating HCl or water [50]. There is thioester formation by the reaction between amino or hydroxyl groups and carboxylic acids in the presence of thiols [47]. There is cross-linking of bifunctional aldehydes (e.g., glutaraldehyde) with chitosan by forming covalent bonds between amino groups [62]. There is sulfonation by the reaction of chitosan with sulfonic acids to form sulfonate esters or sulfonamides [69]. Glycosylation by reducing sugars reacts with chitosan to form glycosidic bonds through condensation [70].
- Lastly, there are coordination reactions like chelation where chitosan amino and hydroxyl groups can coordinate with metal ions (e.g., Cu^2+^, Zn^2+^, Fe^3+^), forming chelate complexes [71]. Chitosan is suited for the sorption of metal ions based in ionic exchange, complexing physical sorption by van der Waals forces and inter- or intracellular trapping [72]. There is cross-linking with divalent or trivalent metal ions (e.g., Fe^3+^ or Ca^2+^) with chitosan, forming a 3D network or gel structure and metal oxide complexes where chitosan can coordinate with metal oxides (e.g., TiO_2_, ZnO) to form hybrid materials [73,74].

As seen, the functionalization of chitosan offers a wide range of derivatives, which are being studied for drug delivery as seen in Table 2. The ability to modify chitosan through various chemical processes is a promising candidate for medical applications. Continued advancements in its functionalization and particle production methods will unlock further potential in innovative therapeutic solutions.

## 4. Preparation Methods of Micro/Nanoparticles of Chitosan

Micro/nanocapsule matrix selection is a critical factor in the success of drug delivery systems, impacting stability, release kinetics, biocompatibility, and targeting. By aligning material properties with the specific requirements of drug delivery and utilizing developments in material science and nanotechnology, researchers can innovate next-generation micro/nanoencapsulation technologies for diverse therapeutic applications. Interdisciplinary research and collaboration will be key to developing safer, more effective, and patient-focused drug delivery systems.

**Figure 5:**
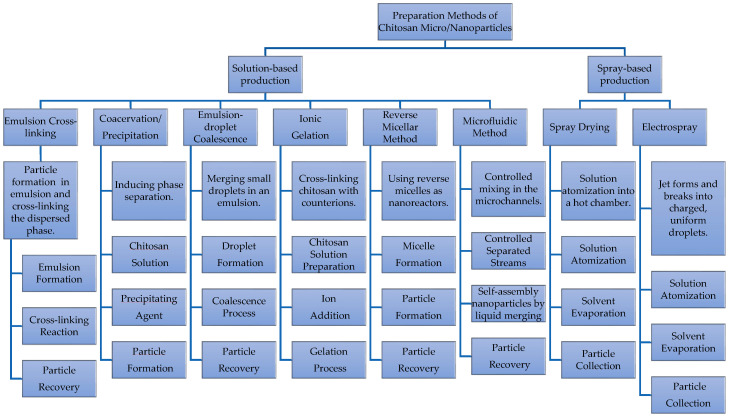
Preparation methods of chitosan micro- and nanoparticles.

Encapsulation methods can be classified as either physical or chemical. Physical methods involve trapping the drug within the encapsulation matrix or adhering it to the surface through adsorption, while chemical methods involve bonding the drug to the encapsulating material or cross-linking it within a network [104]. The properties of both the drug and the encapsulation material, such as solubility, molecular size, and stability, play a crucial role in capsule design. Hydrophobic drugs typically encapsulate better in lipophilic capsules, while hydrophilic drugs perform better in hydrophilic capsules [105]. Larger molecules may pose challenges in encapsulation, and interactions such as covalent bonding, ionic interactions, and hydrogen bonding between the drug and capsule have an influence [106]. Therefore, various capsule (sometimes indistinctly named particle, gel, or sphere) production methods have been developed, such as those solution- and spray-based productions as represented in Figure 5. These include emulsion cross-linking, solvent evaporation, coacervation, precipitation, coalescence, ionic gelation, the reverse micellar method, microfluidic methods, spray drying, and the electrospray method. The chosen method depends on factors such as desired particle size, thermal and chemical stability of the drug, reproducibility, and the stability of the final product.

### 4.1. Solution-Based Production

#### 4.1.1. Emulsion Cross-Linking

The emulsion cross-linking method begins by forming a water-in-oil (W/O) emulsion. The droplets of an aqueous chitosan solution (water-based) are dispersed in an oil phase. The oil phase typically contains a surfactant, which stabilizes the emulsion by preventing the droplets from coalescing or merging. Once the emulsion is formed, a cross-linking agent is added. The cross-linking agent chemically reacts with the chitosan, creating bonds between the chitosan molecules within each droplet. This hardens the droplets, transforming them into solid microspheres. The control of particle size is led by the size of the droplets in the emulsion, more cross-linking produces denser and sometimes smaller microspheres, and faster stirring generally leads to smaller droplets, and hence smaller microspheres [19,107,108].

#### 4.1.2. Ionic Gelation

The complexation between oppositely charged chitosan and tripolyphosphate (TPP) to prepare micro/nanocapsules is simple under mild processing conditions. This method leverages electrostatic interactions for reversible physical cross-linking, avoiding emulsifying agents, which are often toxic to organisms. Chitosan is a positively charged polymer added dropwise to a TPP solution negatively charged polyanion. The electrostatic interaction between them leads the ionic gelation, forming spherical particles. Even though several factors affect the nanoparticle properties, encapsulation efficiency and release rates of the drugs, a higher molecular weight and degree of deacetylation improve encapsulation efficiency and reduce drug release rates. The concentration of chitosan and drugs being encapsulates affects the efficiency and rate of drug release and lower pH levels and higher TPP concentration and increases cross-linking time, leading to slower drug release [72,109,110].

#### 4.1.3. Coacervation/Precipitation

This method for preparing chitosan microspheres uses the properties of chitosan for it to become insoluble in alkaline conditions, or in the presence of oppositely charged biopolymers and the intermolecular electrostatic interactions that lead to the formation of a macroscopic phase separation, which allows the formation of coacervate droplets. Chitosan is soluble in acidic solutions but becomes insoluble in alkaline conditions or oppositely charged biopolymers. Additionally, when a chitosan solution comes into contact with an alcohol, such as methanol, the alcohol dehydrates the chitosan chains. This dehydration reduces the hydrophilic interactions between the chains, causing them to collapse and aggregate, leading to the formation of coacervate droplets [111]. When a chitosan solution comes into contact with an alkaline substance like sodium hydroxide or with alcohols like methanol, coacervate droplets (small, dense liquid droplets) are formed, and also gum arabic–chitosan [112], soy globulin–chitosan [106], xanthan–chitosan [106], zein–chitosan [113], and milk proteins–chitosan [114]. This happens because the chitosan precipitates out of the solution when the pH rises, leading to droplet formation. The chitosan solution might be sprayed through a compressed air nozzle, which breaks the solution into small droplets. These droplets are then exposed to the alkaline solution, leading to the formation of solid microspheres. After droplet formation, the microspheres are separated from the solution through filtration or centrifugation. They are washed to remove any residual chemicals, leaving behind purified chitosan microspheres. The size of the microspheres can be adjusted by varying the air pressure or the diameter of the nozzle used to create the droplets. Higher air pressure or smaller nozzle diameter typically produces smaller microspheres. Cross-linking agents can be used to harden the microspheres further to control the rate at which the encapsulated drug is released. By adjusting the degree of cross-linking, the release profile can be tuned. An alternative technique by using the sodium sulfate method is also possible; sodium sulfate is added to an acidic chitosan solution under stirring and ultrasonication. This leads to the formation of microspheres with enhanced stability in acidic environments. This method does not rely on the alkaline pH for chitosan precipitation but instead uses ionic interaction to form the microspheres [115].

#### 4.1.4. Emulsion-Droplet Coalescence Method

The emulsion-droplet coalescence method combines principles from emulsion cross-linking and precipitation methods. During the emulsion preparation, two separate stable water-in-oil emulsions are created; one emulsion contains an aqueous chitosan solution with the drug and the other emulsion contains the chitosan in a sodium hydroxide solution. The two emulsions are combined under high-speed stirring, causing random collisions between droplets from each emulsion. When droplets from different emulsions collide, they coalesce, leading to chitosan precipitation and droplet formation. This method increases drug loading compared to simple emulsion cross-linking. This technique lets amino groups be free and the electrostatic interactions between chitosan and the drug enhance encapsulation [18].

#### 4.1.5. Reverse Micellar Method

Reverse micelles are composed of water, oil, and the surfactant; the surfactant creates a monolayer around the small water core, creating microscopic domains of water and oil separated by surfactant-rich films [63]. A surfactant is dissolved in an organic solvent to form reverse micelles and the aqueous chitosan solution with the drug is added to the reverse micelle system with constant stirring to maintain an optically transparent microemulsion. A cross-linking agent like glutaraldehyde is added to the mixture and stirred overnight. The organic solvent is evaporated and the remaining transparent dry mass is contained in the nanoparticles. The dry mass is dispersed in water and the surfactant is precipitated out using a suitable salt. The mixture is centrifuged to separate the surfactant and the supernatant containing the nanoparticles. These systems are dynamic with micellar droplets constantly undergoing coalescence and re-separation. This dynamic nature is advantageous for nonreactor function. Reverse micelles are typically 1 to 10 nm and monodisperse [63,116].

#### 4.1.6. Microfluidic Method

Chitosan and cross-linking agents are mixed in microchannels, leading to the formation of uniform droplets. Chitosan is injected into a microfluidic device along with a cross-linker. Due to the controlled mixing in the microchannels, uniform chitosan particles are formed and collected. The device operates by hydrodynamic flow focusing, where controlled mixing occurs primarily through interfacial diffusion [117]. This method is used for pH-triggered self-assembly of chitosan and for loading hydrophobic drugs like paclitaxel into nanoparticles. The system could be a T-shaped polydimethylsiloxane (PDMS) device with three inlets and one outlet. Controlled flows are achieved using micropumps, allowing the precise manipulation of flow rates and mixing time. The central flow (containing chitosan at pH 5.5) is squeezed between two side streams (containing water at pH 9.0) to create a narrow stream of the middle solution. This focusing ensures rapid interfacial diffusion between the solutions, which is key for controlling the self-assembly of chitosan nanoparticles. Chitosan molecules self-assemble when mixed with water at a neutral pH (7.4). By adjusting the flow rates of chitosan and water streams, the mixing time can be controlled to remain in the millisecond range. If the mixing time is shorter than the aggregation time, nanoparticles are kinetically locked into smaller, more monodisperse particles compared to bulk synthesis methods. Lower flow ratios (faster mixing) result in smaller nanoparticles with higher drug loading, while higher flow ratios lead to larger particles and decreased loading efficiency [118,119].

### 4.2. Spray-Based Method Production

#### 4.2.1. Spray-Drying Method

Spray drying is a common and efficient method for creating dry powders, granules, or microspheres from mixtures of drugs and excipients (substances that assist in drug delivery) [120]. Chitosan is first dissolved in an aqueous solution, commonly of acetic acid. The drug to be encapsulated can either be dissolved or dispersed in this chitosan solution, depending on its solubility. Additionally, a cross-linking agent may be added to the solution to enhance the structural stability of the final particles. The prepared solution is then fed into a spray dryer, where it is atomized (broken into fine droplets) by a nozzle. The atomized droplets are introduced into a stream of hot air, which rapidly evaporates the solvent (in this case, water and acetic acid). As the solvent evaporates, the chitosan and drug solidify into tiny, free-flowing particles. The size of the resulting particles can be precisely controlled by adjusting the (1) nozzle size, due to smaller nozzles producing finer droplets, leading to smaller particles; (2) spray flow rate, as the speeds at which the solution is sprayed can influence droplet size and drying time; (3) atomization pressure, due to higher pressure then smaller droplets; (4) inlet air temperature, so higher temperatures accelerate solvent evaporation, which can influence particle formation; (5) extent of cross-linking, as more cross-linking typically results in denser and potentially smaller particles [3,121,122].

#### 4.2.2. Electrospray Method

Chitosan is dissolved in suitable aqueous solvents with the drug incorporated and a cross-linking agent is placed as the aqueous collector bath (counter-electrode) [15]. During the electrospray method, the chitosan solution is pumped towards the needle exit, which is connected to a high voltage. The electric pull overcomes the surface tension of the meniscus at the tip of the needle, forming a pointy meniscus called the Taylor cone [123]. When the electric field is strong enough, a thin jet is expelled from the tip of the Taylor cone [124]. This jet breaks up into finely charged homogeneous-sized droplets. As the droplets move away from the nozzle towards the collector bath, solvent evaporation initiates, leading to the partial or complete drying of the charged droplets [125]. These droplets or nanoparticles are moved by the electric field toward the counter-electrode or collection bath. Upon contact with the cross-linker, the cross-linking process is initiated. These nanoparticles contain both the cross-linked chitosan matrix and the encapsulated drug.

## 5. Manufacturing Challenges in Encapsulation Efficiency and Drug Loading

Each production method for chitosan-based micro- and nanocapsules encounters unique challenges that influence encapsulation efficiency and drug loading capacity. For instance, electrospraying chitosan solutions requires precise control over voltage, flow rates, microfluidics, and the rheological properties of the solution to achieve narrow-size-distribution particles. However, this method suffers from low throughput, making it less practical for large-scale production. Scaling up is particularly challenging due to the need for high voltage, consistent microfluidic performance, and carefully designed electrodes to maintain reproducibility. The production of homogeneous nanocapsules in scaled-up systems has been achieved by using organic polymeric solutions [126] but not in polymeric aqueous solutions. Spray drying achieving consistent particle size and encapsulation efficiency is challenging, as the droplet size is significantly larger than in the electrospray method. Spray drying may also lead to drug loss during production [127]. Emulsion and reverse micellar methods involve multiple phases and require careful control over solvent removal and surfactant concentrations. Ensuring that the droplets remain stable during emulsification and processing is key to successful encapsulation [18,108,128,129]. Coacervation can result in variable micro/nanocapsule size and morphology, particularly when transitioning from small-scale to large-scale production. Temperature control and solvent management are critical for consistency [105,106,114,115]. Ionic gelation relies on the interaction between chitosan and anionic agents to form capsules. The challenge lies in controlling the gelation process to produce micro/nanocapsules with uniform size and drug loading. Scaling up this process can lead to issues with uniformity and reproducibility [108,110,130].

The encapsulation efficiency and drug loading capacity depend on the production of chitosan-based capsules, which are largely determined by the interactions between the drug and the encapsulating matrix. In solution-based encapsulation methods, where encapsulation occurs in liquid media, a notable challenge is that the concentration of reagents decreases over time as more capsules are produced. This depletion leads to variability in the drug-to-matrix ratio, causing inconsistencies in encapsulation efficiency and the surface characteristics of the capsules. As the process continues, the mother mixture becomes less concentrated in active ingredients, leading to differences in the final composition of the capsules [131]. On the other hand, spray-drying and microfluidic techniques offer more uniform results. In these methods, individual droplets are produced and dried in situ, ensuring that each micro/nanocapsule has a consistent composition from batch to batch, as the mixing and drying occur simultaneously.

However, drug loading can be achieved through incorporation during particle formation and post-formation incubation, where the drug is loaded into pre-formed particles. Nonetheless, achieving a balance between high encapsulation efficiency and the structural integrity of the particles is challenged.

The composition of the precursor solution plays a critical role in improving encapsulation, as it helps stabilize the micro/nanocapsule and prevents premature leakage of the active ingredient. The selection of encapsulating materials should be aligned with the specific application and drug properties. Adding stabilizing agents or other excipients can enhance encapsulation by strengthening the interaction between the drug and the micro/nanocapsule. It should be considered that introducing too many additives can lead to unwanted chemical interactions, potentially compromising both drug loading and controlled release. Careful design is essential, from choosing the appropriate encapsulating materials to optimizing the formulation for the intended use, in order to achieve efficient drug delivery with consistent performance [132].

## 6. Targeted Delivery

**Figure 6:**
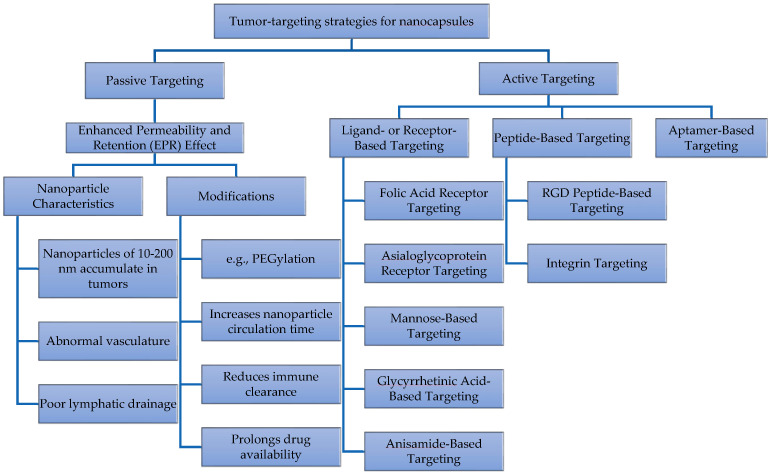
Examples of targeted strategies for drug delivery systems.

Chitosan-based encapsulation has emerged as a promising approach for targeted drug delivery to specific tissues or cells by passive and active targeting [133] as shown in Figure 6. During passive targeting, chitosan nano/microcapsules naturally accumulate in specific tissues or organs based on their physiological properties. For example, in tumors, the Enhanced Permeability and Retention (EPR) effect allows capsules between 10 and 200 nm to penetrate and stay within tumor sites due to abnormal tumor vasculature and poor lymphatic drainage [134]. Various studies have demonstrated that chitosan-based nanoparticles, such as dextran–doxorubicin [13] and paclitaxel-loaded [9] systems, enhance tumor targeting and improve drug efficacy. Modifying capsule size, shape, and surface properties (e.g., PEGylation) can influence their distribution, metabolism, and clearance, increasing their circulation time and reducing immune system removal [135]. For example, polyethylene glycol (PEG)-coated nanoparticles showed prolonged drug availability and reduced liver accumulation compared to uncoated nanoparticles [9].

Passive targeting using the EPR effect enhances drug accumulation in tumors, but it does not always ensure that drugs reach the specific cellular sites needed for effective therapy [133]. To address this, active targeting strategies involving nanoparticles modified with specific receptors, ligands, peptides, and aptamers are being developed to improve cancer specificity, reduce side effects, and enhance drug delivery efficiency [136]. Several targeting strategies are highlighted:

- Folic acid receptors are used to target cancer cells with overexpressed folic receptors, facilitating endocytosis and delivery into the cytoplasm [94].
- Asialoglycoprotein receptors are highly expressed in liver cells [137].
- Metal–organic nanoparticles modified with lactobionic acid improve drug accumulation in hepatocellular carcinoma [138].
- Mannose-based nanoparticles are used for endocytosis and to target macrophages, assisting in immune response regulation [139].
- Glycyrrhetinic acid is used in hepatocyte-targeting ligand functionalization [140].
- Thymoquinone lipidic core nanocapsules with anisamide polymethacrylate shells are used for colon cancer cells overexpressing sigma receptors [141].
- Arginine–glycine–aspartic acid (RGD) peptides target tumor and tumor-endothelial cells in vivo [142], and there is Integrin targeting in human cell lines [143].
- Aptamer-modified nanoparticles target specific cancer antigens, such as the prostate-specific membrane antigen [30,144].

## 7. Mechanisms of Controlled Release and Sustained Release

Lastly, once the micro/nanocapsule is on the target site, the release profile (e.g., sustained or controlled release) depends on the cross-linking density, and for balancing encapsulation efficiency and drug release rates, particle size for desired release kinetics and environmental conditions regards the pH value. Usually, drug release from the chitosan matrix is controlled by swelling [39,107,122,145,146].

Therefore, higher cross-linking of chitosan nanoparticles lowers encapsulation efficiency and reduces swelling but it slows down drug release. pH and ionic strength of the media affect drug solubility and matrix swelling. A more acidic or basic medium can accelerate the swelling–shrinking of the chitosan matrix, leading to faster drug release. Porosity allows for faster diffusion and release, while a smoother surface slows drug release. Increased surface area by reducing particle size enhances drug release [147,148,149].

As mentioned previously, drug release from chitosan-based particles involves several complex mechanisms, determined by factors mentioned before. (1) In surface-dependent release, the drug is released directly from or adsorbed onto the surface of the particles. This often results in an initial burst effect, characterized by rapid release when the system encounters the release medium. And by increasing the cross-linking density, the burst release can be reduced, but this may impact the overall encapsulation efficiency [150]. (2) In diffusion through the swollen chitosan matrix, water penetrates, causing the polymer matrix to swell, allowing the drug to diffuse out. Initially, release is slow but accelerates as the chitosan swells. The molecular weight of chitosan and particle size can significantly affect this process. Smaller particles, with a larger surface area, typically release drugs faster [151]. (3) In erosion release, the chitosan matrix degrades over time, providing a sustained release profile. This mechanism allows for drug release over an extended period, which is useful for long-term therapeutic applications [152].

## 8. Conclusions

In conclusion, chitosan presents significant potential as a versatile material for drug delivery due to its tunable properties and functionalization capabilities. However, designing an effective drug delivery system requires a careful evaluation of the selected chitosan derivative based on the specific properties of the drug and the chosen targeting strategy. The selection process must consider factors such as the molecular weight, degree of deacetylation, solubility, and responsiveness to environmental triggers (e.g., pH, temperature, or enzymes). By aligning the functionalized chitosan with the characteristics of the drug and the desired targeting strategy, whether passive or active, optimized therapeutic outcomes can be achieved. Continued research into chitosan-based systems will further enhance their efficacy, stability, and specificity, making them increasingly valuable for a wide range of medical applications.

## Figures and Tables

**Table 1:** Clinical trials regarding chitosan usage in their formulations (https://www.clinicaltrials.gov/search?term=Chitosan&viewType=Table accessed on 5 September 2024).

NCT Number	Study Title	Phase	Conditions	Study Status	Study Results	Last Update Posted	Location
NCT05893888	Study of PRV111 and PRV211 in Subjects with Oral Squamous Cell Carcinoma	1, 2	Oral Squamous Cell Carcinoma	Recruiting	NO	2024	United States
NCT06525363	Clinical Assessment of Chitosan Nanoparticles on Periodontal Problems Post Steroidal Inhalation in Asthmatic Patients	1	Periodontal Diseases	Not yet recruiting	NO	2024	
NCT06513039	Evaluation of Erythropoietin on Alveolar Ridge Preservation	4	Alveolar Bone Loss	Recruiting	NO	2024	Egypt
NCT06491992	Chitosan Phonophresis on Cervical IN Smartphone Addicted Users	NA	Cervical Pain	Recruiting	NO	2024	Egypt
NCT06492811	Hydrogel Containing Cascade Catalytic Enzymes in the Treatment of Diabetic Wounds	2	Diabetic Wound	Active, not recruiting	NO	2024	China
NCT05661708	Chitosan Powder in Loop Electrosurgical Excision Procedure	4	Vaginal Bleeding	Completed	NO	2024	Turkey
NCT05970731	Topical Drug Delivery for Treatment for PASC Hyposmia	2	Post Acute Sequelae COVID-19 Hyposmia	Recruiting	NO	2024	United States
NCT06457360	Comparison of Chitosan, Ankaferd and Tranexamic Acid in Dental Extraction in Liver Pre-Transplant Children	1	Liver Cirrhosis	Completed	NO	2024	Egypt
NCT06426199	Chitosan-Hyaluronate Gel Mixture Vs Hyaluronic for Internal Derangement	4	TMJ Disk Disorder	Recruiting	NO	2024	Egypt
NCT06396013	Chitosan Nanocrystalline Qinteng Huoxue Runji Ointment Therapy for Psoriasis With Blood Stasis Syndrome	1	Plaque Psoriasis	Not yet recruiting	NO	2024	
NCT04887389	Cariostatic and Remineralizing Effects of Three Different Dental Varnishes	4	Dental Caries	Completed	NO	2024	Egypt
NCT03993678	Intratumoral Injection of IP-001 Following Thermal Ablation in Patients With Advanced Solid Tumors	1, 2	Advanced Solid Tumors	Recruiting	NO	2024	Switzerland
NCT06135259	Topical Erythropoietin Hydrogel in Management of Oral Lichen Planus	3	Oral Lichen Planus	Not yet recruiting	NO	2024	
NCT06200415	Glucosamine Sulphate Versus Ginger in Non-Surgical Periodontal Therapy	2	Periodontal Pocket	Completed	NO	2024	Egypt
NCT06175676	Thermosensitive Hydroxybutyl Chitosan and 5-fluorouracil Assisted Endoscopic Dacryocystorhinostomy in the Treatment of Chronic Dacryocystitis	1	Chronic Dacryocystitis	Completed	NO	2023	China
NCT06172023	Evaluation of Antimicrobial Efficacy and Postoperative Pain After Using Silver Nanoparticles and Chitosan Nanoparticles Against Enterococcus Faecalis and Candida Albicans Biofilm	NA	Endodontic Disease	Completed	NO	2023	Egypt
NCT06039774	Mangostin Hydrogel Film With Chitosan Alginate Base for Recurrent Aphthous Stomatitis (RAS)	2	Recurrent Aphthous Stomatitis	Recruiting	NO	2023	Indonesia
NCT06072716	The Anti-fungal Effect of Miconazol Versus Miconazol-Loaded Chitosan Nanoparticles	1	Oral Thrush	Completed	NO	2023	Egypt
NCT05875506	Efficacy of Ozone Gel, Doxycycline Saturated Chitosan Dressing Versus Alveogyl in Pain Alleviation and Healing of Alveolar Osteitis	NA	Osteitis|Dry Socket	Recruiting	NO	2023	Egypt
NCT05333211	Ortho-RÂ^®^ for Rotator Cuff Repair Compared With Standard of Care Rotator Cuff Repair Without Ortho-RÂ^®^	1, 2	Rotator Cuff Tears	Unknown	NO	2023	United States
NCT05683782	Erythropoietin Gel as an Adjunct to Split-Thickness Apically Positioned Flap in Augmentation of Attached Gingiva	4	Erythropoietin|Recession	Recruiting	NO	2023	Egypt
NCT04005872	Clinical and Radiographic Evaluation of Nano Silver Fluoride Versus Calcium Hydroxide in Indirect Pulp Treatment	NA	Deep Caries	Completed	NO	2022	Egypt
NCT04219358	Topical Application of Imiquimod Gel at Different Concentrations in Actinic Cheilitis	1	Actinic Cheilitis	Terminated	NO	2022	Brazil
NCT05591872	Low Dose Heparin Factorial Trial	3	Radial Artery Occlusion	Recruiting	NO	2022	Pakistan
NCT04308863	Evaluation of Chitosan Scaffold and Mineral Trioxide Aggregate Pulpotomy in Mature Permanent Molars With Irreversible Pulpitis	2	Pulpitis—Irreversible	Completed	NO	2022	Egypt
NCT04481945	Evaluation of Antimicrobial Efficacy and Adaptability of Bioceramic Sealer Containing Nanoparticles	4	Endodontic Disease	Completed	NO	2022	Egypt
NCT03712371	Study of Chitosan for Pharmacologic Manipulation of AGE (Advanced Glycation Endproducts) Levels in Prostate Cancer Patients	1, 2	Prostate Cancer	Terminated	NO	2021	United States
NCT05079802	Clinical & Radiographic Evaluation of LSTR in Non-vital Primary Molars Using Two Different Vehicles	3	Resorption of Tooth or Root	Unknown	NO	2021	
NCT03186261	Antibacterial Effect of Nano Silver Fluoride vs Chlorhexidine on Occlusal Carious Molars Treated With Partial Caries Removal Technique	3	Dental Caries	Completed	YES	2021	Egypt
NCT04365270	Antibacterial Effect and Clinical Performance of Chitosan Modified Glass Ionomer	3	Caries	Completed	NO	2021	Egypt
NCT02789033	Efficacy of the Combination of Isosorbide Dinitrate Spray and Chitosan in Diabetic Foot Ulcers	3	Diabetic Foot Ulcers	Completed	YES	2021	
NCT04319575	Comparison of Triple Antibiotic Paste With Combination of Chitosan and Calcium Hydroxide as a Root Canal Disinfectant	2, 3	Periapical Abscess	Unknown	NO	2020	India
NCT02591017	Comparison of Oral Morphine Versus Nasal Ketamine Spray With Chitosan in Cancer Pain Outpatients	3	Cancer Pain	Terminated	NO	2018	Switzerland
NCT03724266	Effect of Using Nanochitosan Versus Calcium Hydroxide as Disinfectant on Pain and Apical Bone Healing	NA	Postoperative Pain|Healing	Unknown	NO	2018	
NCT03719261	Assessment of Pain and Antibacterial Activity of Chitosan Versus Sodium Hypochlorite as Irrigant in Infected Canal	2, 3	Postoperative Pain	Unknown	NO	2018	
NCT03588351	Chitosan, Chitosan Nanoparticles, and Chlorhexidine Gluconate, as Intra Canal Medicaments in Primary Teeth	NA	Necrotic Pulp	Unknown	NO	2018	Egypt
NCT03202446	Randomized Clinical Trial Evaluating the Use of the Laser-Assisted Immunotherapy (LIT/inCVAX) in Advanced Breast Cancer	3	Breast Cancer	Terminated	NO	2018	Peru
NCT03259217	Clinical Application of Mesenchymal Stem Cells Seeded in Chitosan Scaffold for Diabetic Foot Ulcers	1	Stem Cell Transplant	Unknown	NO	2017	
NCT03188289	Microbial Growth in the Suture Thread, After Application of Different Antiseptic Gels in Mandibular Third Molars Extraction	4	Oral Surgery	Completed	NO	2017	
NCT02858297	Glucosamine as a Novel Adjunctive Therapy in Oral Lichen Planus	4	Oral Lichen Planus	Completed	NO	2016	
NCT01950546	Nanosilver Fluoride to Prevent Dental Biofilms Growth	1	Dental Caries	Completed	NO	2015	Brazil
NCT00806962	Phase 1 Norwalk Vaccine Study	1	Norovirus	Completed	NO	2015	United States
NCT01753752	Evaluation of the Corneal Residence Time of Chitosan-N-acetylcysteine Eye Drops in Patients With Dry Eye Syndrome After Single and Multiple Instillation	2	Dry Eye Syndrome	Completed	NO	2013	Austria
NCT01275547	The Analgesic Effect of Combined Treatment With Intranasal S-ketamine and Intranasal Midazolam	2, 3	Analgesia	Completed	NO	2013	Switzerland

**Table 2:** Chitosan derivatives.

Chitosan Derivative	* Solubility	* Biodegradability	* Antimicrobial Activity	* Drug Delivery Efficiency	Applications	Advantages	Disadvantages	Ref.
*N,N,N*-Trimethyl Chitosan (TMC)	H	M	H	H	Mucoadhesive Drug Delivery	Excellent mucoadhesion, enhanced antimicrobial activity	Potential cytotoxicity at H concentrations	[75]
Carboxymethyl Chitosan (CMC)	H	M	M	M	Wound Healing, Drug Delivery	Good water solubility, pH-sensitive	Decreased mechanical strength	[21,51]
Chitosan Oligosaccharides (COSs)	H	H	H	M	Antimicrobial, Anti-inflammatory	Low molecular weight, good tissue penetration	Rapid degradation	[71]
Chitosan–PEG Conjugates	H	M	L	H	Extended Drug Circulation	Enhanced solubility, improved biocompatibility	Reduced antimicrobial activity	[76,77]
N-Phthaloyl Chitosan (PhCh)	L	M	L	M	Hydrophobic Drug Delivery	Good compatibility with hydrophobic drugs	Complex synthesis, potential biocompatibility issues	[78]
Chitosan–TPP Nanoparticles	H	H	M	H	Targeted Drug Delivery	Controlled release, H encapsulation efficiency	Potential for aggregation, stability issues	[15]
Quaternary Ammonium Chitosan (QAC)	H	L	H	M	Antimicrobial Coatings	Broad-spectrum antimicrobial activity	Cytotoxicity at H concentrations	[52]
Sulfonated Chitosan (S-Chitosan)	H	M	L	M	Cardiovascular Applications	Anticoagulant, anti-inflammatory	Reduced mechanical strength, risk of over-anticoagulation	[59]
Chitosan–Gelatin Blends	M	H	M	M	Tissue Engineering, Wound Healing	Enhanced mechanical strength, biocompatible	Variability depending on gelatin content	[79]
Chitosan–Lauric Acid (Chitosan-LA)	L	M	H	H	Targeted Drug Delivery	Enhanced antimicrobial properties, good hydrophobic drug encapsulation	Complex synthesis, potential cytotoxicity	[56]
Chitosan–Cysteine Conjugate	M	H	M	H	Mucoadhesive Drug Delivery	Improved drug absorption, sustained release	Potential for disulfide cross-linking, cytotoxicity	[80]
Chitosan–g-PNIPAM	M	M	M	H	Smart Drug Delivery	Temperature-sensitive release	Complex synthesis, incomplete release	[81]
Hydroxybutyl Chitosan (HBC)/Graphene Oxide	H	H	L	M	Tissue Engineering, Wound Healing	Enhanced flexibility, good film-forming properties	Potential loss of antimicrobial activity	[82]
Chitosan–Lactate	H	M	L	M	Wound Healing, Drug Delivery	Good solubility in neutral pH, promotes cell proliferation	Reduced antimicrobial activity	[83]
Chitosan–Montmorillonite Nanocomposites	L	M	M	M	Packaging, Wound Healing	Improved mechanical and thermal stability	Complex synthesis, variability in properties	[84]
Chitosan–Glycerol Blends	M	H	L	L	Biodegradable Packaging, Wound Healing	Good flexibility, biocompatible	Reduced mechanical strength, loss of antimicrobial activity	[85]
Chitosan–Stearic Acid (Chitosan-SA)	L	M	H	H	Targeted Drug Delivery	Good hydrophobic drug encapsulation, antimicrobial	Complex synthesis, cytotoxicity	[55]
Chitosan–Collagen Blends	M	H	M	M	Tissue Engineering, Wound Healing	Enhanced mechanical properties, promotes cell adhesion	Variability depending on collagen content	[86]
Chitosan–Graft-acrylic Acid	H	M	M	M	Hydrogels, Drug Delivery	pH-sensitive, controlled release	Complex synthesis, instability in extreme pH	[87]
Chitosan–Benzoic Acid	L	M	H	M	Antimicrobial Coatings	Enhanced antimicrobial properties	Complex synthesis, cytotoxicity	[88]
N-Methylene Phosphonic Chitosan	H	M	L	L	Environmental Applications	Excellent chelation properties, low toxicity	Reduced antimicrobial activity	[89]
Chitosan–Hydroxyapatite Nanocomposites	L	H	M	M	Bone Tissue Engineering	Supports bone regeneration, good mechanical properties	Complex synthesis, variability in properties	[90]
Chitosan–Methylglyoxal Conjugates	M	M	H	M	Antimicrobial Coatings	Effective against resistant strains	Potential cytotoxicity, reduced stability	[91]
Chitosan–EDTA	H	M	L	L	Environmental Applications	Excellent chelation properties, biocompatible	Reduced antimicrobial activity	[92]
Chitosan–Diethylaminoethyl (DEAE)	H	M	M	H	Mucoadhesive Drug Delivery	Enhanced drug delivery, biocompatible	Cytotoxicity at H concentrations	[91]
Chitosan–Poly lactic-*co*-glycolic Acid (PLGA)	M	H	M	H	Drug Delivery, Tissue Engineering	Extended circulation time, controlled release	Complex synthesis, instability in extreme pH	[93,94]
Chitosan–g-PVA	H	M	M	M	Wound Dressings, Tissue Engineering	Enhanced mechanical strength, good film-forming properties	Complex synthesis, instability in extreme pH	[95]
Chitosan–Bioactive Glass Composites	L	H	M	M	Bone Tissue Engineering	Supports bone regeneration, good mechanical properties	Complex synthesis, variability in properties	[96]
Chitosan–Alginate Blends	M	H	M	M	Wound Healing, Drug Delivery	Biocompatible, good mechanical properties	Variability depending on blend ratio	[18]
Chitosan–Polylactic Acid	M	H	M	H	Drug Delivery, Tissue Engineering	Controlled release, extended circulation time	Complex synthesis, instability in extreme pH	[97]
Chitosan–Poly (ε-caprolactone	L	M	M	H	Tissue Engineering, Drug Delivery	Good mechanical properties, controlled release	Complex synthesis, reduced biodegradability	[98]
Chitosan–g- Polystyrene	L	M	L	M	Adsorption of Heavy Metal Ions, Packaging	Good mechanical properties, potential for coatings	Complex synthesis, reduced biodegradability	[99]
Chitosan–Grafted-poly(ethylene oxide)	H	M	L	H	Extended Drug Circulation	Improved solubility, extended circulation time	Reduced antimicrobial activity	[100]
Chitosan–Beta-Cyclodextrin Conjugates	H	M	M	H	Targeted Drug Delivery	Enhanced solubility, good drug encapsulation	Complex synthesis, reduced stability	[79]
Chitosan–Dextran Sulfate Complexes	H	M	M	M	Wound Healing, Drug Delivery	Biocompatible, controlled release	Complex synthesis, instability in extreme pH	[101]
Chitosan–Lysozyme Conjugates	M	M	H	M	Antimicrobial Coatings	Broad-spectrum antimicrobial activity	Complex synthesis, potential cytotoxicity	[102]
Chitosan–Sodium Alginate Complexes	M	H	M	M	Wound Healing, Drug Delivery	Biocompatible, good mechanical properties	Variability depending on blend ratio	[18]
Chitosan–Carboxymethyl Cellulose	H	M	M	M	Wound Healing, Drug Delivery	Good mechanical properties, biocompatible	Variability depending on blend ratio	[103]

* H: High, M: Medium, L: Low.

## Data Availability

The data presented in this study are available on request from the corresponding author.
